# Human iPSC-Derived Renal Cells Change Their Immunogenic Properties during Maturation: Implications for Regenerative Therapies

**DOI:** 10.3390/cells11081328

**Published:** 2022-04-13

**Authors:** Bella Rossbach, Krithika Hariharan, Nancy Mah, Su-Jun Oh, Hans-Dieter Volk, Petra Reinke, Andreas Kurtz

**Affiliations:** 1 Berlin Institute of Health Center for Regenerative Therapies (BCRT), Charité Universitätsmedizin Berlin, 13353 Berlin, Germany; krithika.hariharan@ibmt.fraunhofer.de (K.H.); idefix83@snu.ac.kr (S.-J.O.); hans-dieter.volk@charite.de (H.-D.V.); petra.reinke@charite.de (P.R.); 2 Fraunhofer Institute for Biomedical Engineering (IBMT), Fraunhofer-Forum Berlin, 10178 Berlin, Germany; nancy.mah@ibmt.fraunhofer.de; 3 Fraunhofer Institute for Biomedical Engineering (IBMT), Fraunhofer Project Center for Stem Cell Processing, 97082 Würzburg, Germany; 4 Institute for Medical Immunology (IMI), Charité Universitätsmedizin Berlin, 13353 Berlin, Germany; 5 Berlin Center for Advanced Therapies (BeCat), Charité Universitätsmedizin Berlin, 13353 Berlin, Germany

**Keywords:** human induced pluripotent stem cells, renal differentiation, cell replacement therapy, HLA barrier

## Abstract

The success of human induced pluripotent stem cell (hiPSC)-based therapy critically depends on understanding and controlling the immunological effects of the hiPSC-derived transplant. While hiPSC-derived cells used for cell therapy are often immature with post-grafting maturation, immunological properties may change, with adverse effects on graft tolerance and control. In the present study, the allogeneic and autologous cellular immunity of hiPSC-derived progenitor and terminally differentiated cells were investigated in vitro. In contrast to allogeneic primary cells, hiPSC-derived early renal progenitors and mature renal epithelial cells are both tolerated not only by autologous but also by allogeneic T cells. These immune-privileged properties result from active immunomodulation and low immune visibility, which decrease during the process of cell maturation. However, autologous and allogeneic natural killer (NK) cell responses are not suppressed by hiPSC-derived renal cells and effectively change NK cell activation status. These findings clearly show a dynamic stage-specific dependency of autologous and allogeneic T and NK cell responses, with consequences for effective cell therapies. The study suggests that hiPSC-derived early progenitors may provide advantageous immune-suppressive properties when applied in cell therapy. The data furthermore indicate a need to suppress NK cell activation in allogeneic as well as autologous settings.

## 1. Introduction

Human induced pluripotent stem cells (hiPSCs) provide an unlimited source material for functionally differentiated cells suitable for use in cell-replacement therapies (CRTs) and tissue engineering [1,2]. Compared to human embryonic stem cells (hESCs), iPSC-technology offers the possibility of personalized autologous CRT and thus might overcome rejection barriers connected to alloimmunogenicity. Surprisingly, transplanted syngeneic murine iPSCs (miPSCs) were shown to be rejected in a T cell-driven manner [3]. Consequently, the immunological effects of clinically applicable hiPSC-derivatives are a major concern. Reprogramming and cultivation-based neoantigens may cause some of these immunological effects; however, despite the development of integration-free reprogramming techniques and xeno-free media, iPSC-derived cells may still invoke a variable response from the immune system [3,4,5]. For example, certain miPSC-derivatives, such as endothelial cells, dermal cells, bone marrow cells, hepatocytes and neuronal cells, were not rejected in syngeneic recipients, whereas transplanted miPSC-derived cardiomyocytes elicited significant levels of T cell infiltration [6,7,8]. In a humanized mouse model, hiPSC-derived retinal pigment epithelial cells (RPECs) were tolerized by autologous reconstituted T cells, whereas differentiated smooth muscle cells (SMCs) were rejected [9]. It was demonstrated that the aberrant expression of Zymogen granule protein 16 (ZG16) induced the immunogenic nature of hiPSC-derived SMCs. Aberrant expression of Zg16, as well as of HORMA domain-containing protein 1 (Hormad1), has already been shown in autologous transplanted miPSCs and deemed responsible for their immunogenic nature [3].

For efficient CRT, cells at different maturation stages may be needed depending on the patient, disease, tissue, desired cell type and the anticipated mode of action. Terminally differentiated cells, such as hiPSC-derived RPECs, might directly replace damaged cells in macular degenerative diseases, whereas further cell proliferation and differentiation post-grafting may be required to rebuild and restore a complex tissue structure, such as the hematopoietic system [10,11]. However, it remains unknown whether the maturation stage of hiPSC-derived cells modulates their immunogenic characteristics and the immunomodulatory properties after transplantation.

In addition, due to the logistical and cost-related challenges of autologous hiPSC-based therapies, the allogeneic off-the-shelf approach is a major focus of research. Efforts are underway to establish an unlimited resource of hiPSC lines that are haploidentical and homozygous for common human leucocyte antigen (HLA) alleles to decrease allogeneic mismatches of the differentiated products [12,13].

In the described study, the kidney was used as a model system to investigate the maturation stage-dependent auto- and allogenicity of hiPSC-derived cells. Renal cells are a relevant example for epithelial cells and kidney replacement therapies are a major clinical need. Different types of renal cells at defined developmental stages can be derived nowadays from hiPSCs [14,15]. These cells could be used therapeutically for CRT in patients suffering from acute kidney injury (AKI) or chronic kidney disease (CKD)—both global health problems with increasing prevalence [16]. Indeed, transplanted miPSCs and miPSC-derived renal progenitors, respectively, were shown to support regeneration processes and improved renal function in immunocompromised and immune-suppressed models of AKI, respectively [17,18].

Induction of intermediate mesoderm cells (IMCs) is the first step in renal differentiation from hiPSCs, followed by epithelialization and specification of the nephron elements, including proximal epithelial cells (PTCs). Damage to PTCs is the leading cause of AKI and subsequently CKD. The immunological responses of human T and natural killer (NK) cells towards hiPSCs and hiPSC-derived IMCs and PTCs were analyzed using sensitive in vitro assays. Moreover, primary urinary cells (pUCs) collected from hiPSC donors were used to directly compare donor-specific allogeneic and autologous responses against hiPSC-derived and non-hiPSC-derived cells in an isogenic setup. Immune phenotypic analysis revealed decreased HLA-ABC and HLA-DR expression in hiPSCs and hiPSC-derived renal cells compared to pUCs. Although allogeneic T cell activation was observed against pUCs, neither autologous nor allogeneic hiPSCs or hiPSC-derived renal cells induced T cell responses. However, hiPSCs and hiPSC-derived renal cells showed susceptibility to NK cells. Active immunomodulatory properties were observed in hiPSCs, IMCs and early-stage PTCs, which may explain the attenuated immune response of the cells even in allogeneic conditions and imply an at least temporary immune-privileged status for hiPSCs and hiPSC-derived precursor cells. Taken together, an immune-privileged status for hiPSC-derived IMCs and PTCs was identified, which declines with cell maturation.

## 2. Materials and Methods

### 2.1. Ethics Statement

All human cells were obtained with informed consent and approval from the Ethics Committee of Charité Universitätsmedizin Berlin, which also covers the derivation and use of hiPSCs (approval number EA4/110/10 and 126/2001).

### 2.2. Human Cells Lines

Two hiPSC lines, BCRTi004-A and BCRTi005-A, were used, generated from primary urinary cells (pUCs) of two healthy female donors using integration-free Sendai virus (SeV) technology [19,20]. Both hiPSC lines are registered at the Human Pluripotent Stem Cell Registry (hPSCreg). Conditional immortalized cell lines were a gift from Dr. Si-Hong Luu. The generation was performed as described previously [21].

### 2.3. Differentiation of hiPSCs into Renal Cell Types

hiPSCs were differentiated into IMCs and into PTCs using a stepwise protocol with slight modifications [14]. Briefly, for IMC differentiation, 4 × 10^4^ hiPSCs/cm^2^ were seeded on Geltrex (Thermo Fisher Scientific, Waltham, MA, USA)-coated plates in TeSR-E8 media (Stem Cell Technologies, Vancouver, Kanada) supplemented with Y27632 (Wako Chemicals USA Inc., Richmond, VA, USA). After two days, mesendoderm differentiation was induced using 5 µM CHIR90221 (Tocris, Bristol, UK) dissolved in complete Advanced RPMI (A-RPMI) composed of basal A-RPMI (Thermo Fisher Scientific) supplemented with 1% GlutaMax (Thermo Fisher Scientific) and 1% Penicillin/Streptomycin solution (Thermo Fisher Scientific). After 36 h, media were switched to intermediate mesoderm induction media for 72 h, composed of complete A-RPMI supplemented with 2 µM Retinoic acid (Stemgent, Cambridge, MA, USA) and Fibroblast growth factor 2 (Peprotech, Cranbury, NJ, USA). IMCs were cultivated for 7 days in complete A-RPMI to obtain PTCs. PTCs were cultivated for another 14 days on Geltrex-coated plates including weekly passages to promote their further maturation (LT-PTCs). Differentiated cells were harvested for analysis and co-culture assays on day 5 (IMCs), day 12 (PTCs) and day 26 (LT-PTCs) post induction.

### 2.4. Human Primary Cells

pUCs from healthy donors were isolated as described previously [22]. After expansion in complete A-RPMI, pUCs were cryopreserved in heat-inactivated fetal calf serum (FCS, Biochrom, Cambridge, UK) supplemented with 10% dimethyl sulfoxide (DMSO, Sigma-Aldrich, St. Louis, MO, USA) until use. Peripheral blood mononuclear cells (PBMCs) from healthy donors and patients were isolated using Biocoll (Merck KGaA, Darmstadt, Germany) density gradient centrifugation. After two washing steps in phosphate-buffered saline w/o calcium and magnesium (PBS w/o Ca and Mg, Thermo Fisher Scientific) an optional erythrocyte lysis (Qiagen, Hilden, Germany) was performed. Obtained PBMCs were resuspended in co-culture media, consisting of KnockOut Dulbecco’s modified eagle medium (KO-DMEM, Thermo Fisher Scientific) supplemented with 20% KnockOut Serum Replacement (KOSR, Thermo Fisher Scientific), 1% GlutaMax, 0.1 mM non-essential amino acids (NEAA, Thermo Fisher Scientific), 1% β-Mercaptoethanol (Thermo Fisher Scientific) or stored in cryopreservation media at −160 °C until use.

### 2.5. Immunofluorescence Staining of Adherent Cells

Cultured cells were first washed with PBS containing Ca and Mg. Afterwards, cells were fixed using Cytofix (BD Biosciences, Franklin Lakes, NJ, USA). Permeabilization was performed using 10% donkey serum (Millipore, Burlington, MA, USA) diluted in Perm/Wash buffer (BD). Primary antibody incubation with NPHS2, SLC22A2, LRP2, Na/K-ATPase (all obtained from Abcam, Cambridge, UK), PAX2 (Life Technologies, Carlsbad, CA, USA), LHX1, AQP1, AQP2 and SLC12A3 (all obtained from Novus Biologicals, Centennial, CO, USA) occurred overnight at 4 degrees (antibody details described in Table S1). After washing three times with Perm/Wash, incubation with fluorescent-labeled secondary antibodies (Thermo Fisher Scientific) was performed in the dark at room temperature. Cells were washed again three times before the final staining of the nuclei using 4′,6-diamidin-2-phenylindol (DAPI, Sigma-Aldrich). Afterwards, DAPI solution was replaced finally with PBS with Ca and Mg. Control samples subjected to the staining procedure, including secondary antibody incubation but excluding primary antibody incubation, were used to assess the specificity of primary antibodies and for background subtraction. Cell images were obtained using the Opera Phenix High Content Screening device (PerkinElmer, Waltham, MA, USA) and final analysis was performed using Columbus Software (version 2.9, PerkinElmer).

### 2.6. Transcriptome Analysis

Samples were harvested using Gentle Dissociation Reagent (for hiPSCs; StemCell Technologies) or Trypsin (Merck) and total RNA was extracted using the Qiagen RNA Mini Kit. cDNA libraries from poly-A-tail enriched RNA were prepared from hiPSCs, IMCs, PTCs, LT-PTCs and pUCs using a TruSeq mRNA sample prep kit v.2 (Illumina, San Diego, CA, USA). Sequence alignment and RNA-Seq analysis: Bcl to fastq conversion was performed using Illumina software (Illumina). Fastq files were aligned against human reference build hg19 provided by the Genome Reference Consortium (GRCh19). Transcript alignment was performed using TopHat. Analysis of differential expression and transcript abundance was performed using Cuffdiff from the Cufflinks analysis package (version 2.1.1). All heatmaps were generated using the gplots package in R-statistical software (version > 3.4). The heatmaps picture the mean FPKM for genes (heatmap rows) for different groups (heatmap columns).

### 2.7. Functional Uptake Assays

Glucose uptake by hiPSCs, hiPSC-derived renal cells and pUCs, respectively, was tested using the fluorescent glucose analog 2-(*N*-(7-Nitrobenz-2-oxa-1,3-diazol-4-yl)Amino)-2-Deoxyglucose (2-NBDG; Thermo Fisher Scientific). Upfront, cells were washed using DMEM without glucose (Gibco). To assess specificity, the SGLT2 inhibitor Dapagliflozin (Thermo Fisher Scientific) was used as a control and respective samples were incubated for 30 min in a final concentration of 0,5 µM Dapagliflozin prior to the assessment. Subsequently, cells were incubated for 30 min with 200 µg/mL 2-NBDG in the presence or absence of Dapagliflozin. For nuclei staining, Hoechst 33342 was added to the samples for the last 15 min.

Endocytosis of albumin was tested using native human serum albumin protein conjugated to FITC (Abcam). Prior incubation with different concentrations of FITC-albumin, hiPSCs, IMCs, PTCs, LT-PTCs and pUCs were washed using serum-free RPMI (Thermo Fisher Scientific), respectively. Afterwards, FITC-albumin was added either in a final concentration of 100 ng/mL or 1000 ng/mL for 60 min. Respective control wells were left untreated. Hoechst 33,342 was added for the last 15 min to visualize cell nuclei.

After a final washing step using, again, either DMEM without glucose or serum-free RPMI, live cell imaging was performed using the Opera Phenix High Content Screener. Data analysis was conducted using Columbus software.

### 2.8. Fluorescence Labeling of PBMCs

T cell proliferation was tracked using fluorescent dye distribution within daughter cell generation. Up to 10^7^ PBMCs were resuspended in PBS w/o Ca and Mg and incubated with either CellTrace Violet (CTV) or a CellTrace carboxyfluorescein succinimidyl ester Kit (CFSE, both Thermo Fisher Scientific) in a final concentration of 5 µM.

### 2.9. Flow Cytometry

For characterization, hiPSCs, IMCs, PTCs and LT-PTCs were harvested at 0, 5, 12 and 26 days post-induction, respectively. Collected cells were permeabilized using Perm2 buffer (BD Biosciences) and further blocked in 10% donkey serum (Merck). Cells were incubated with unlabeled primary antibodies, PAX2, LHX1, AQP1 (Proteintech Group, Rosemont, IL, USA) and Na/K-ATPase, respectively, and afterwards stained with secondary conjugated anti-donkey antibodies (Thermo Fisher Scientific). For immune phenotype analysis of hiPSCs, hiPSC-derived renal cells and pUCs, conjugated antibodies against HLA-ABC and HLA-DR (both BioLegend, San Diego, CA, USA) were used for cell surface staining. Harvested PBMCs from the supernatants of co-cultures were collected and further stained for vitality (L/D Blue, Invitrogen, Waltham, MA, USA) and the surface markers CD3, CD4, CD8, CD25, CD40, CD45RA, CD56, CD69, CD80, CD86, CD95, CCR7 and HLA-DR (BioLegend, eBiosciences (San Diego, CA, USA)), BD Biosciences, Beckman Coulter Diagnostics (Brea, CA, USA); antibody details described in Table S1). For intracellular staining of FOXP3 and CTLA4 (both BD Biosciences), PBMCs were permeabilized using a Foxp3 Transcription Buffer Set (eBiosciences). Flow cytometry analysis was performed using a LSR-Fortessa device and cell sorting was performed using the Aria SORP (both obtained from BD Biosciences). Results were analyzed with FlowJo 887 (Tree Star, San Francisco, CA, USA). For analysis of PBMC responses, individual gate settings were performed based on the negative (unstimulated) and positive (SEB stimulated) control individually for each donor.

### 2.10. Immune Cell Proliferation Assay

The stimulator cells, hiPSCs, IMCs, PTCs, LT-PTCs and pUCs, were seeded into 24 wells (5 × 10^4^ per well) in either co-culture media supplemented with Y27632 (hiPSCs) or complete A-RPMI supplemented with Y27632 (renal differentiated cells, pUCs). After attachment, cells were stimulated with IFNγ for 48 h with 25 ng/mL, as previously described [23]. Then, 2 × 10^5^ CTV labeled PBMCs were added to irradiated (30 Gy) stimulator cells. After 7 days of co-culture, non-adherent PBMCs were harvested and lymphocyte proliferation was assessed by CTV tracking via flow cytometry. For further T cell stimulation, either allogeneic B cells in a 1:10 ratio or SEB (Sigma-Aldrich) in a final concentration of 100 ng/mL was added. For analysis of immunosuppressive capacities, co-cultured hiPSCs, IMCs, PTCs and LT-PTCs were used non-irradiated.

### 2.11. Cytokine Detection

Supernatants of PBMC co-cultures and monocultures of hiPSCs, IMCs, PTCs, LT-PTCs and pUCs, respectively, were taken on days 1 and 3 and analyzed for transforming growth factor beta (TGFβ) using an enzyme-linked immunosorbent assay (ELISA; BioLegend) and Interferon-gamma (IFNγ) and tumor necrosis factor-alpha (TNFα) using a multiplex bead-based assay (Meso Scale Discovery) according to the manufacturer´s protocol. For the determination of total TGF-β, supernatants were treated with acidification solution before measurement, while the assessment of free active TGFβ was obtained without prior acidification. ELISA samples were measured using a plate reader (SpectraMax 340PC) at 450 nm and at 570 nm to exclude non-specific background staining. Multiplex samples were detected using the plate reader MESO QuickPlex SQ 120.

### 2.12. Statistical Analysis

Results are shown as means; in certain cases the mean ± standard error of the mean (SEM) is shown. Statistical differences between two groups were assessed using a nonparametric Mann–Whitney test. A comparison of more than two groups was performed using Kruskal–Wallis non-parametric one-way analysis of variance (ANOVA) with Dunn´s post hoc test. A value of *p* < 0.05 was considered statistically significant. Graph preparation and statistical analysis were performed using GraphPad Prism 5 (GraphPad Software Inc., San Diego, CA, USA).

## 3. Results

### 3.1. Generation and Maintenance of hiPSC-Derived Renal Cells

**Figure 1:**
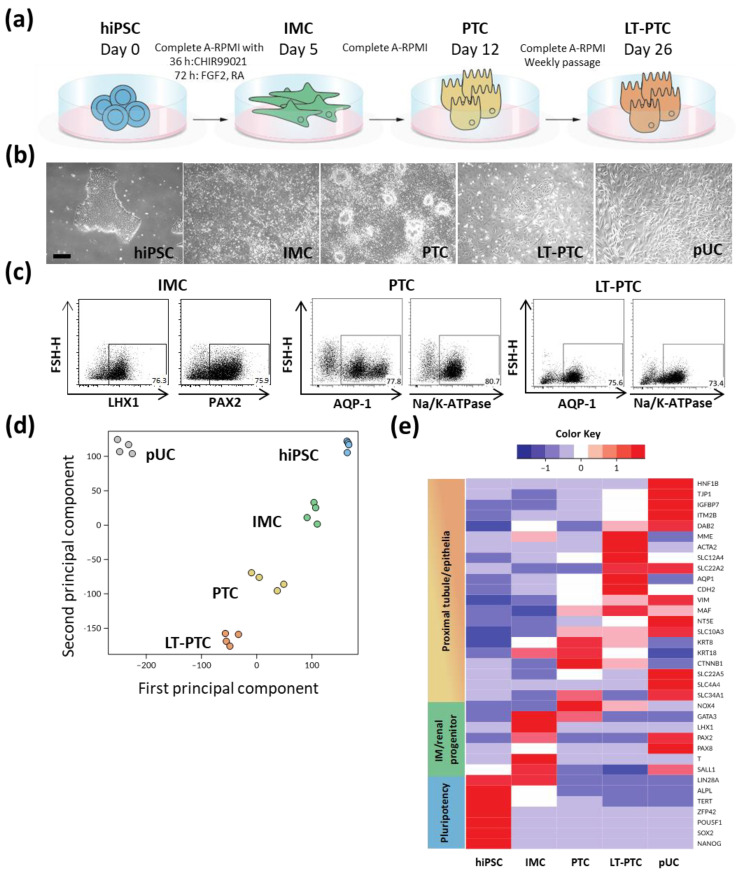
Reprogrammed pUCs differentiate with high efficiency into renal progenitors and into PTCs, which can be stably maintained in vitro. (**a**) hiPSCs, generated from pUCs, were differentiated into IMCs and PTCs using a stepwise protocol. Differentiated PTCs were further cultivated for two additional weeks (LT-PTCs). (**b**) Differentiated cells were examined by phase-contrast microscopy and showed stage-specific cell morphology. (**c**) Cell stage-specific proteins for IMCs—PAX2 and LHX1—and for PTCs—AQP1 and Na/K-ATPase—were analyzed by flow cytometry to determine differentiation efficiencies. Transcriptome analysis revealed (**d**) differential clustering as depicted in a PCA plot and (**e**) stage-specific gene expression in hiPSCs, hiPSC-derived IMCs, PTCs, LT-PTCs and primary pUCs. Data are representative of biological duplicates per cell type and cell line. The expression values (FPKM) of each gene (row) are normalized by a row z-score. Scale bar: 100 μm.

Isolated primary cells from urinary sediments of healthy donors include mostly exfoliated renal tubular cells and urinary tract epithelial cells [24]. Expanded pUCs showed heterogeneous morphologies consisting of cuboid and spindle-like cells and were used to generate the urinary-cell derived hiPSC lines BCRTi004-A and BCRTi005-A (Figure 1a and Figure S1) [19,20]. Differentiation of BCRTi004-A and BCRTi005-A consistently generated IMCs and PTCs via a stepwise protocol (Figure 1a,b) [14]. Typical cobblestone morphologies were observed in PTCs and maintained in long-term cultivated proximal tubular cells (LT-PTCs) for at least 26 days (Figure 1b). Analysis of cell type-specific marker expression by flow cytometry revealed up to 80% differentiation efficiencies (Figure 1c). IMCs were defined by the expression of the IM-specific markers LIM homeobox 1 (LHX1) and Paired box 2 (PAX2). PTCs were assessed by the occurrence of Aquaporin 1 (AQP1) and Sodium-potassium-adenosine triphosphatase (Na/K-ATPase), which remained stably expressed after continued cultivation for 14 days on Geltrex (Figure 1b,c and Figure S2). In comparison, undifferentiated hiPSCs did not show expression of IMC- and PTC-specific markers (Figure S3).

Additional immunostaining revealed as well proximal tubule identity of PTCs, LT-PTCs and pUCs due to high expression of Solute carrier family 22 member 2 (SLC22A2) and LDL receptor-related protein 2 (LRP2), respectively (Figure S4), and due to the functional uptake ability of FITC-albumin and the glucose analog 2-NBDG (Figure S5). Comparatively, specific marker expression for podocytes (Podocin), distal tubules (SLC12A3) or collecting ducts (AQP2) was not detectable in IMCs, PTCs, LT-PTCs or pUCs (Figure S4).

Principal component analysis (PCA) of RNA-sequencing data for hiPSCs, hiPSC-derived renal cells and pUCs revealed differential clustering of the respective cell types (Figure 1d).

For more in-depth analysis of differentiation and maturation progression, stage-specific marker gene expression was compared (Figure 1e). The data confirmed decreased expression of pluripotency-associated genes such as Octamer-binding transcription factor 4 (OCT4), Sex-determining region Y-box 2 (SOX2) and Telomerase reverse transcriptase (TERT) in IMCs, PTCs and LT-PTCs. The markers LHX1, PAX2, PAX8 and Gata binding protein 3 (GATA3), as well as the mesendoderm marker Brachyury (T), were specifically upregulated in IMCs. Increased expression of PTC markers, such as the membrane transport proteins SLC10A3, SLC12A4, AQP1, the epithelial markers Catenin beta 1 (CTNNB1), Keratin 8 (KRT8), KRT18 and the adhesion molecule Cadherin 2 (CDH2), occurred in the differentiated PTCs and showed stable expression during long-term cultivation in LT-PTCs. Together, these results indicate that hiPSC-derived renal cells faithfully recapitulate the stages of IMCs and PTCs during kidney development and further maturation progression in the LT-PTCs.

### 3.2. Immune Phenotype of hiPSCs and hiPSC-Derived Renal Cells

**Figure 2:**
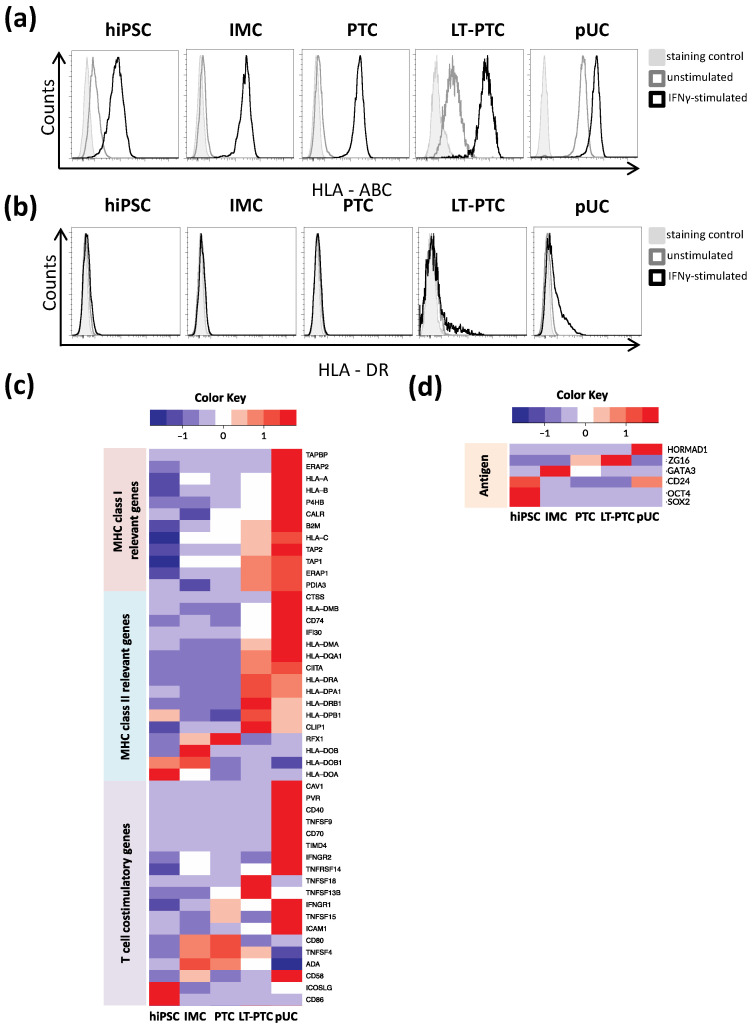
Immune phenotypes of hiPSCs and hiPSC-derived renal cells are less mature compared to pUCs but are regulated by a pro-inflammatory environment. hiPSCs, differentiated IMCs, PTCs and LT-PTCs were analyzed for HLA-ABC and HLA-DR molecules on the cell surface by flow cytometry. Adult somatic pUCs were used in comparison. Representative dot plots are shown for (**a**) HLA-ABC and (**b**) HLA-DR under homeostatic and under pro-inflammatory conditions induced by IFNγ stimulation. HLA-ABC molecule numbers were low on the surface of hiPSCs, IMCs and PTCs but were increased on LT-PTCs, whereas pUCs expressed moderate levels. HLA-DR molecules were only detectable in LT-PTCs and pUCs after IFNγ stimulation. (**c**,**d**) Transcriptome profiles were used to analyze expression of (**c**) MHC class I, MHC class II, T cell co-stimulatory molecules and (**d**) common immunogenic antigens in hiPSCs, and hiPSC-derived renal cells and expression intensities were compared to somatic pUCs. Data are representative of three IFNγ stimulated biological replicates per cell type. The expression values (FPKM) of each gene (row) are normalized by a row z-score.

Expression of MHC class I (HLA-ABC) and MHC class II (e.g., HLA-DR) is essential for the recognition of antigens by cluster of differentiation (CD)8^+^ T cells and CD4^+^ T cells, respectively. The presence of HLA-ABC and HLA-DR molecules on the surface of hiPSCs, IMCs, PTCs, LT-PTCs and pUCs was assessed to elucidate their capacity to elicit T cell responses. The cells were stimulated by interferon-gamma (IFNγ), which induces upregulation of HLA-ABC and HLA-DR expression in pro-inflammatory environments triggered by infiltrating leukocytes, to simulate damaged tissue usually faced by cells in CRT [23]. Under homeostatic conditions, hiPSCs, IMCs and PTCs expressed very low levels of HLA-ABC (Figure 2a). After stimulation with IFNγ, HLA-ABC expression was strongly upregulated in the hiPSCs, IMCs and PTCs. HLA-DR was not detectable on these cell types even after IFNγ treatment (Figure 2b). Interestingly, LT-PTCs in comparison showed increased HLA-ABC expression already under homeostatic conditions, and expression levels further increased upon IFNγ stimulation. Additionally, HLA-DR expression in LT-PTCs was induced by IFNγ stimulation. As expected for maturated renal cells, pUCs expressed consistently high levels of HLA-ABC [25], which was further elevated upon IFNγ stimulation. Additionally, HLA-DR expression was induced by IFNγ.

To gain a better understanding of the immune phenotype of IFNγ-treated hiPSCs, IMCs, PTCs, LT-PTCs and pUCs, transcriptomes were analyzed for the expression of genes related to MHC class I, MHC class II and T cell co-stimulatory factors (Figure 2c). MHC class I-related genes, such as the respective polymorphic α-chains, non-polymorphic β2-microglobulin (β2M), the transporter associated with antigen processing 1 (TAP1) and TAP2, were expressed at lower levels in hiPSCs compared to pUCs and upregulated with progressing differentiation. Immune maturation continued in LT-PTCs after specification of the proximal tubular phenotype in PTCs. MHC class II genes showed variable expression throughout the developmental stages. However, the Class II major histocompatibility complex transactivator (CIITA), crucial for MHC class II expression, was detectable in LT-PTCs and pUCs only. Common T cell co-stimulatory molecules such as CD80 and CD86 were detectable in hiPSCs, IMCs, PTCs, LT-PTCs as well as in pUCs. Other co-stimulatory factors, such as CD40, CD70, and tumor necrosis factor ligand superfamily member 9 (TNFSF9), showed highest expression in pUCs, whereas expression in hiPSCs and renal derivatives was markedly low and did not show differentiation stage associations.

Next, we examined the transcript expression of potentially immunogenic antigens, such as SOX2, OCT4, HORMAD1, ZG16, CD24 and GATA3, which were previously described to elicit immune responses leading to the rejection of miPSCs, hiPSCs and their derivatives by antigen-specific T cells in preclinical models (Figure 2d) [3,9,26,27,28]. OCT4 showed high expression in hiPSCs, while residual OCT4 expression was strongly reduced in IMCs, PTCs and LT-PTCs. CD24 was highly expressed in hiPSCs, showing downregulation during renal differentiation, and expression almost disappeared in LT-PTCs. The transcription factor GATA3 is selectively expressed during the embryogenesis of the human kidney and is thus highly transcribed in IMCs [29]. HORMAD1 was not detectable at any stage during renal differentiation and showed expression only in pUCs, although at a very low level (data not shown), whereas ZG16 was marginally detectable in PTCs and LT-PTCs.

In summary, donor-identical pUCs show a more immunogenic phenotype than hiPSCs and hiPSC-derived renal cell types; however, phenotypic immunogenicity moderately increases at later cellular states in LT-PTCs. Moreover, potentially immunogenic antigens show expected cell type-specific expression patterns.

### 3.3. Autologous T Cell Response against hiPSC-Derived Renal Cells

**Figure 3:**
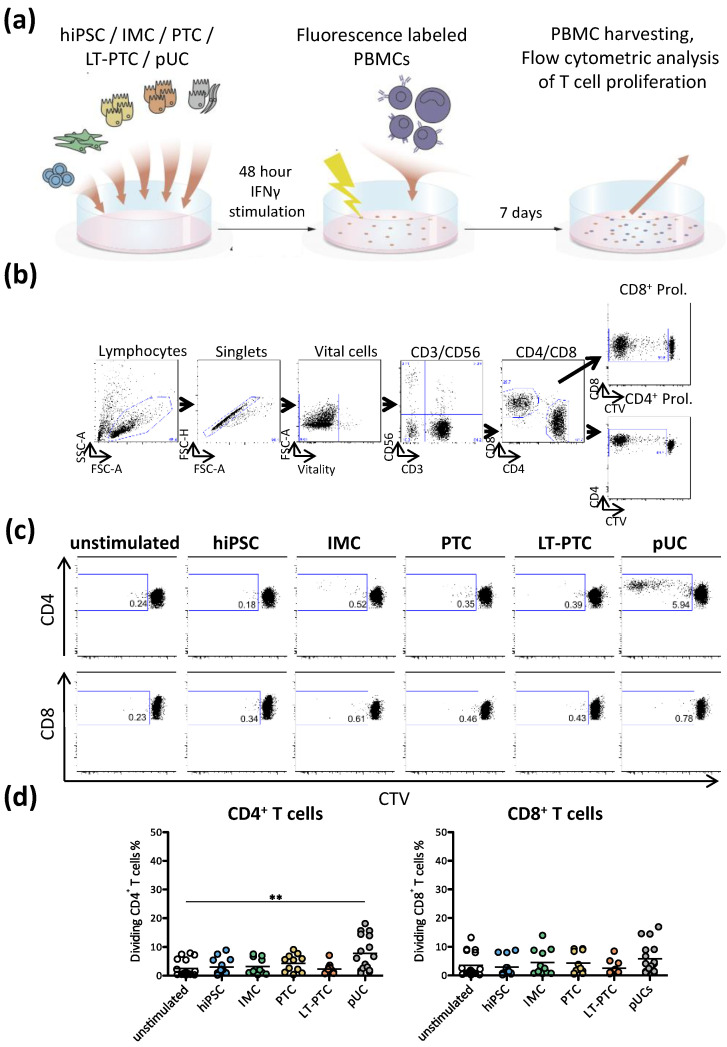
Autologous derived IMCs, PTCs and LT-PTCs do not elicit T cell proliferation. Co-cultures of IFNγ-stimulated hiPSCs, IMCs, PTCs, LT-PTCs and pUCs with autologous PBMCs, respectively, were performed for 7 days and T cell proliferation was tracked using a fluorescence-based method, as shown in (**a**). Proliferation of CD4^+^ and CD8^+^ T cells was assessed using flow cytometry, and gating was performed as depicted in (**b**), as shown for the SEB stimulated sample. (**c**) Representative plots indicate the absence of proliferation of CD4^+^ and CD8^+^ T cells, respectively, when stimulated with autologous hiPSCs and hiPSC-derived IMCs, PTCs and LT-PTCs. (**d**) The graph depicts a summary of 11–18 independent experiments showing the mean. Statistical analysis was performed using one-way ANOVA followed by Dunn´s post hoc test. ** *p* < 0.01.

Although the analysis of the immune phenotype of hiPSC-derived renal cells revealed low expression of genes with regard to the MHC class I and MHC class II complexes compared to primary somatic cells, prediction of immunogenicity based on transcript and protein expression patterns alone is not possible, as immune responses can be triggered by very low levels of HLA-peptide complexes. In vitro one-way mixed lymphocyte reactions (one-way MLR) were performed to elucidate T cell responses triggered by autologous hiPSCs and hiPSC-derived renal cells. Peripheral blood mononuclear cells (PBMCs) isolated from pUC donors—the same used for the generation of the hiPSC lines BCRTi004-A and BCRTi005-A, respectively—were used (Figure 3a). Thus, all stimulator cells (hiPSCs, pUCs) shared identical HLA genotypes. CD4^+^ and CD8^+^ T cell proliferation was monitored after co-cultivation of fluorescent-labeled autologous PBMCs with hiPSCs, IMCs, PTCs, LT-PTCs and pUCs, respectively (Figure 3b). Unstimulated control was performed using only PBMCs cultivated without any additional cell type or stimulant to assess unspecific background T cell proliferation. A negligible fraction of CD4^+^ and CD8^+^ T cells proliferated in response to stimulation by any of the autologous hiPSC-derived renal cell types or undifferentiated hiPSCs (Figure 3c). In summary, renal cells differentiated from hiPSCs did not show susceptibility to autologous T cells (Figure 3d).

### 3.4. Allogeneic T Cell Response against hiPSC-Derived Renal Cells

**Figure 4:**
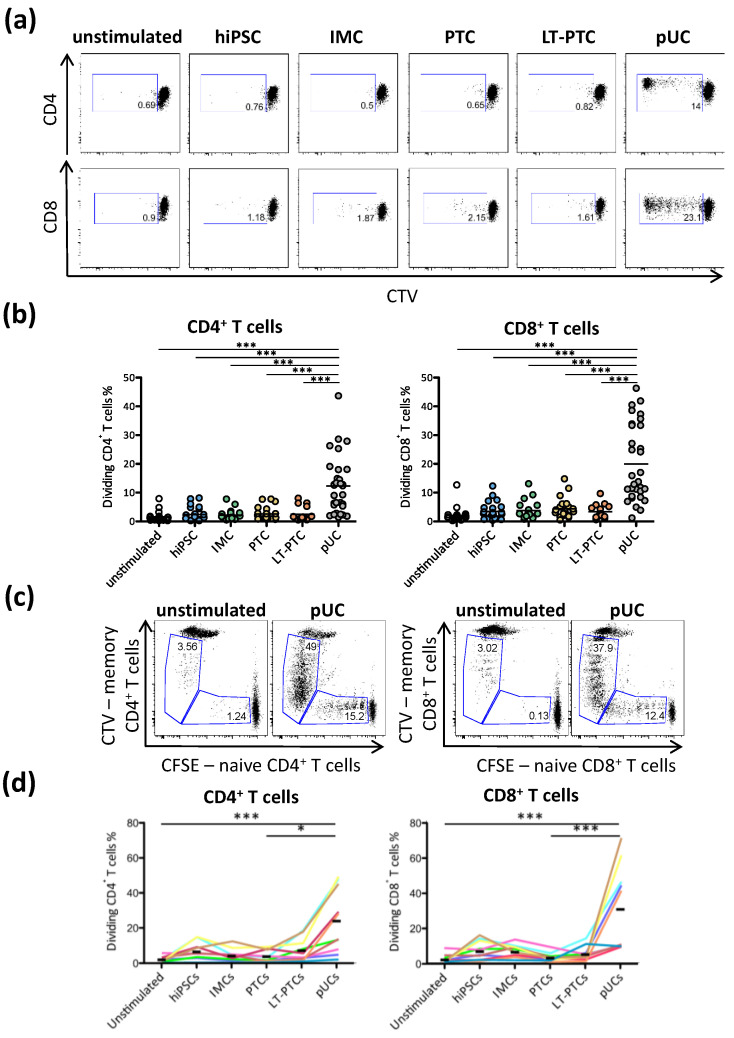
HLA-mismatched, allogeneic hiPSCs, IMCs, PTCs and LT-PTCs fail to induce proliferation of T cells from healthy donors and patients with diabetic nephropathy. (**a**) Representative dot plots did not indicate induction of T cell proliferation by allogeneic hiPSCs, IMCs, PTCs and LT-PTCs, whereas allogeneic HLA-identical primordial pUCs elicited strong CD4^+^ and CD8^+^ T cell proliferation. (**b**) Summary of performed co-cultures of hiPSCs, hiPSC-derived renal cells and pUCs with PBMCs of healthy donors for 20 to 31 independent experiments. (**c**) Healthy donors were analyzed for the pre-existence of allogeneic memory T cells against primordial pUCs. Before co-culture of PBMCs with pUCs, naïve T cells were separated and labeled with CFSE, whereas residual PBMCs that contained memory T cells, among others, were stained with CTV. Independent tracking of naïve T cells and memory T cells after seven days of co-culture using flow cytometry revealed proliferation of naïve and memory T cells in parallel, as depicted. (**d**) PBMCs of patients with diabetic nephropathy were co-cultured with allogeneic hiPSCs, IMCs, PTCs, LT-PTCs and pUCs. Significant T cell proliferation was obtained against pUCs, whereas hiPSCs and hiPSC-derived renal cells did not induce allogeneic T cell proliferation. Each colored line (*n* = 10) represents data from a unique co-culture experiment of patient´s PBMCs with one set of hiPSCs, renal differentiated hiPSCs and corresponding pUCs. (**c**,**d**) Statistical analysis was performed using one-way ANOVA followed by Dunn´s post hoc test. * *p* < 0.05; *** *p* < 0.001.

Allogeneic off-the-shelf hiPSC lines could represent an attractive source for clinical applications. To assess the immunogenicity of allogeneic hiPSCs and hiPSC-derived renal cells, PBMCs donated by unrelated, unmatched healthy donors were co-cultured with hiPSCs, IMCs, PTCs, LT-PTCs and pUCs, respectively, in one-way MLRs (Figure 3a,b). Available HLA types of allogeneic PBMCs from healthy donors showed at most one shared HLA-A/B/DR allele with the hiPSC lines used (Figure S6). For the tracking of unspecific T cell proliferation, unstimulated PBMCs without any stimulant were tested and defined as a negative control. Tracking of CD4^+^ and CD8^+^ T cells in PBMCs showed high proliferation response against allogeneic pUCs (Figure 4a,b). Analysis of involved T cell subpopulations revealed pre-formed memory T cells as well as naïve T cells specific against allogeneic pUCs (Figure 4c and Figure S7). In contrast, although HLA-identical with allogeneic pUCs, hiPSCs, IMCs, PTCs and LT-PTCs did not induce allogeneic T cell proliferation in any of the PBMCs originating from unmatched healthy individuals. Further expression analysis of the activation marker HLA-DR on CD4^+^ and CD8^+^ T cells after 7 days of co-cultivation confirmed reduced immunogenicity of allogeneic hiPSCs and renal descendants compared to allogeneic pUCs (Figure S8). Furthermore, the level of released pro-inflammatory tumor necrosis factor-alpha (TNF𝛼) on day 3 was only elevated after co-cultivation with allogeneic pUCs (Figure S9).

In clinical situations, patients undergoing kidney CRT may present increased memory T cell levels and variability, as with diabetic patients, who have confirmed elevated numbers of allogeneic memory T cells compared to healthy individuals [30]. We, therefore, used PBMCs from patients with diabetic nephropathy to study the rejection characteristics of allogeneic hiPSC-derived renal cells and pUCs (Table S2). Although pUCs, again, induced strong T cell proliferation, hiPSCs and hiPSC-derived renal cells, sharing the same HLA type as pUCs, did not (Figure 4d). Furthermore, direct comparison of all experimental groups confirmed that T cell responses between autologous and allogeneic hiPSCs, IMCs, PTCs and LT-PTCs were essentially indistinguishable in comparison to pUCs (Figure S10). In conclusion, hiPSCs and hiPSC-derived renal cells showed immune-privileged properties. They neither induced proliferation and activation of allogeneic naïve T cells nor of pre-formed allogeneic memory T cells, while HLA-identical pUCs elicited robust allogeneic T cell responses.

### 3.5. Immunomodulatory Properties of hiPSCs, IMCs, PTCs and LT-PTCs

**Figure 5:**
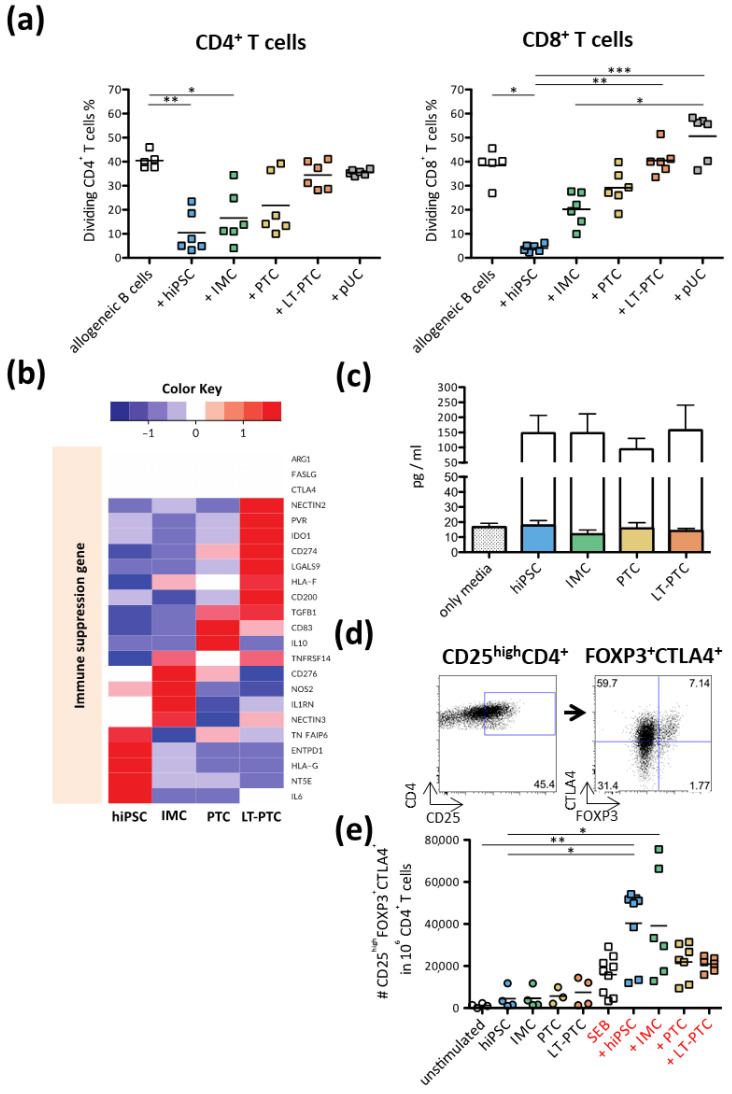
hiPSCs, IMCs and PTCs but not LT-PTCs exhibit T cell immunosuppressive properties. (**a**) HLA-mismatched hiPSCs, IMCs, PTCs, LT-PTCs and pUCs were added as third parties to allogeneic B cell-stimulated PBMCs. T cell proliferation was inhibited by hiPSCs, IMCs and PTCs, while LT-PTCs and pUCs failed to suppress stimulated T cell proliferation. (**b**) Transcriptome profiles of hiPSCs and hiPSC-derived renal cells were analyzed for the expression of common immunosuppressive molecules, and expression intensities were compared within the different cell types. Data are based on three IFNγ-stimulated biological replicates for each cell type. (**c**) ELISA was performed to quantify TGFβ secreted by hiPSCs and hiPSC-derived renal cells. Elevated levels of latent forms of TGFβ were detected in the supernatants of hiPSCs, IMCs, PTCs and LT-PTCs (colored bars indicate active TGFβ, white bars indicate latent TGFβ). (**d**) hiPSCs and hiPSC-derived renal cells were analyzed for the capacity to polarize conventional CD4^+^ into a regulatory phenotype. After co-culture of PBMCs with hiPSCs, IMCs, PTCs, LT-PTCs and pUCs, respectively, regulatory T cells were identified as CD25^high^FOXP3^+^CTLA4^+^ using flow cytometry. (**e**) The total number of CD4^+^ T cells with a regulatory phenotype was assessed after co-culture of PBMCs with hiPSCs/hiPSC-derived renal cells alone, respectively, and after the addition of the polyclonal T cell stimulator SEB (*n* = 4–9). For the statistical analysis, one-way ANOVA was performed with subsequent Dunn´s post hoc testing. * *p* < 0.05; ** *p* < 0.01; *** *p* < 0.001. The expression values (FPKM) of each gene (row) are normalized by a row z-score.

To analyze the nature of disabled allogeneic T cell response, active immunomodulatory properties were studied in hiPSCs and hiPSC-derived renal cell types. Thus, hiPSCs, IMCs, PTCs, LT-PTCs and pUCs were added, respectively, as third parties to allogeneic mixed lymphocyte reactions (MLRs), respectively. Activated B cells expressing CD40, CD80 and CD86 were used as allogeneic stimulators (Figure S11). Activated B cells induced, on average, 40% responder CD4^+^ and CD8^+^ T cell proliferation (Figure 5a). The addition of hiPSCs highly suppressed allogeneic CD4^+^ and CD8^+^ T cell proliferation. Furthermore, the addition of IMCs and PTCs, respectively, also reduced T cell proliferation after allogeneic B cell stimulation, but to a lower extent than hiPSCs. In comparison, LT-PTCs did not exhibit active immunomodulatory functions, while the addition of allogeneic pUCs to the allo-MLR even increased allogeneic T cell proliferation.

We used transcriptome data to identify potential candidates of immunosuppressive molecules secreted by hiPSCs and hiPSC-derived renal cells (Figure 5b). Remarkably, we did not detect in hiPSCs and hiPSC-derived renal cells at the mRNA level the common immunosuppressive molecules Arginase 1 (ARG1) and Fas ligand (FASLG), which were previously reported to be expressed in PSCs and to induce tolerance against allogeneic immunity [31,32]. Moreover, expression of T cell inhibitory receptor cytotoxic T-lymphocyte associated protein 4 (CTLA4) and anti-inflammatory cytokine interleukin 10 (IL-10) was not observed. Instead, transcriptome data revealed an association of renal cell maturation with RNA levels of the known immune suppression and immune escape genes Transforming growth factor-beta 1 (TGFβ), Indoleamin-2,3-dioxygenase (IDO1), CD274, the non-classical MHC class I molecule HLA-F and the ligands Poliovirus receptor (PVR) and Nectin cell adhesion molecule 2 (NECTIN2).

TGFβ is a pleiotropic polypeptide regulating multiple physiological processes, including T cell growth and development [33]. It has been demonstrated that TGFβ acts as a potent inducer of Forkhead box P3 (FOXP3), leading to de novo generation of induced regulatory T cells (iTregs) [34]. Using an enzyme-linked immunosorbent assay (ELISA), we confirmed protein secretion of TGFβ by hiPSCs, IMCs, PTCs and LT-PTCs. However, the obtained data also demonstrated that secreted TGFβ was still in its latent and thus inactive form (Figure 5c). We further wanted to investigate whether the increased expression of latent TGFβ by hiPSCs and hiPSC-derived renal cells in an allogeneic setting could lead to the conversion of conventional CD4^+^CD25^-^ T cells into iTregs.

After stimulation of PBMCs with allogeneic hiPSCs, IMCs, PTCs and LT-PTCs, respectively, the total number of CD4^+^ iTreg cells marked by the co-expression of CD25^high^CTLA4^+^FOXP3^+^ was identified (Figure 5d). We did not observe increased numbers of FOXP3^+^ Tregs in any of the co-culture experiments (Figure 5e). In contrast, the additional presence of the polyclonal T cell activator Staphylococcal enterotoxin B (SEB) in one-way MLR with hiPSCs and IMCs, respectively, led to significantly higher numbers of CD25^high^CTLA4^+^FOXP3^+^ cells compared to SEB alone or unstimulated controls. In conclusion, hiPSCs, IMCs and PTCs possess active immunomodulatory capacities. The immunosuppressive impact declines with progression of differentiation, since LT-PTCs and adult pUCs did not exhibit active anti-proliferative effects on stimulated allogeneic T cells.

### 3.6. Autologous and Allogeneic NK Cell Responses to hiPSCs and hiPSC-Derived Renal Cells

**Figure 6:**
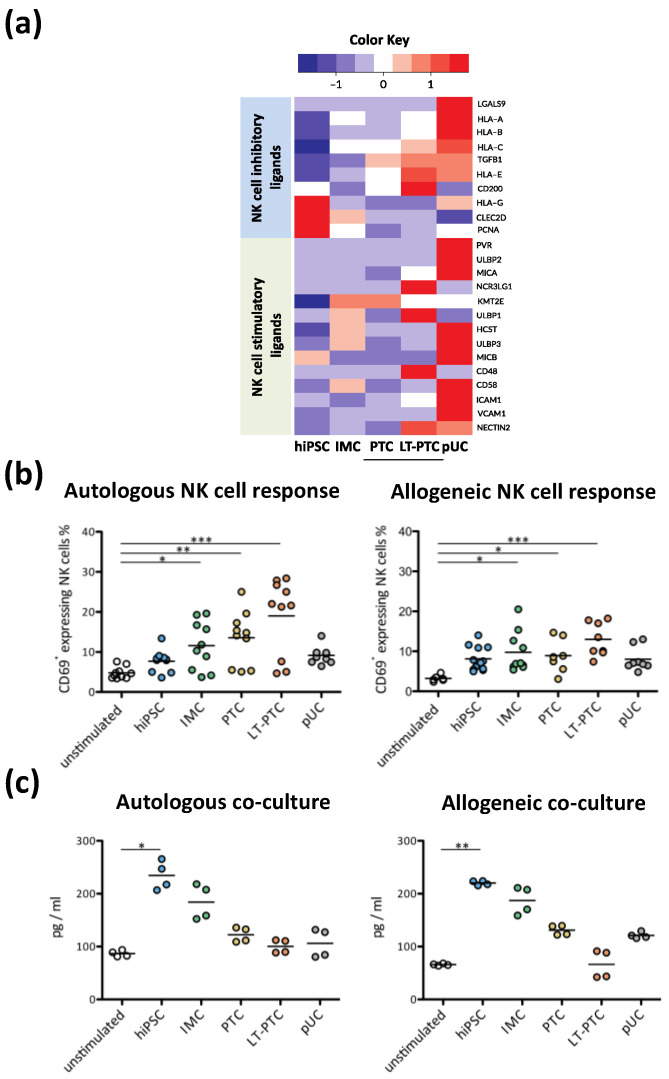
hiPSCs and hiPSC-derived renal cells are prone to autologous and allogeneic NK cells. (**a**) Transcriptome analysis was used to identify expression intensities of NK cell stimulatory and NK cell inhibitory ligands on hiPSCs, IMCs, PTCs, LT-PTCs and pUCs for comparison. Three biological IFNγ-stimulated replicates were used for each cell type. (**b**) Co-cultures were performed with autologous and allogeneic PBMCs from healthy donors. After one day of co-culture, PBMCs were harvested and NK cells were analyzed for CD69 using flow cytometry. NK cell activation was increased when exposed to autologous (*n* = 6–12) and allogeneic (*n* = 10) IMCs, PTCs and LT-PTCs. (**c**) After 1 day of co-culture, the accumulation of IFNγ in the co-culture media was analyzed by multiplex assay. Obtained data revealed significant elevations of IFNγ amounts in the co-culture of PBMCs with autologous and allogeneic hiPSCs, respectively (*n* = 4). The expression values (FPKM) of each gene (row) are normalized by a row z-score. Statistical analysis was performed using one-way ANOVA followed by Dunn´s post hoc test. * *p* < 0.05; ** *p* < 0.01; *** *p* < 0.001.

Previous reports described contradictory results regarding the susceptibility of pluripotent stem cells to NK cells [23,35,36]. We, therefore, examined the sensitivity of NK cells to hiPSCs and the hiPSC-derived renal cells. NK cells express several stimulatory receptors, such as Natural killer group 2 (NKG2D) receptors, which promote cytotoxic and inflammatory activation and might lead to the elimination of ligand-expressing cells [37]. Transcriptome analysis of hiPSCs, IMCs, PTCs, LT-PTCs and pUCs revealed expression of the NKG2D ligands stress-related MHC class I polypeptide-related sequence (MIC) A, MICB, UL16 binding protein (ULBP) 2 and ULBP3, with highest expression in pUCs (Figure 6a). Expression of NK cell inhibitory ligands, such as HLA-E, HLA-G, CD200 and C-type lectin domain family 2 member D (CLEC2D), however, were increased in hiPSCs and in LT-PTCs, respectively, in comparison to pUCs.

To assess the activation status of NK cells, we performed a one-way MLR of PBMCs with autologous and allogeneic hiPSCs, IMCs, PTCs, LT-PTCs and pUCs, respectively. NK cells showed significantly increased levels of the activation marker CD69 on the cell surface after 24 h of exposure to autologous as well as to allogeneic IMCs, PTCs and LT-PTCs, respectively (Figure 6b).

In contrast to NK cell activation, analysis of the pro-inflammatory cytokine IFNγ in co-culture supernatants revealed the highest levels, with autologous as well as allogeneic undifferentiated hiPSCs (Figure 6c). The observed NK cell activation and IFNγ response were thus comparable for autologous and allogeneic cell sources. In contrast to previous studies, our analysis revealed only a minor impact of IFNγ pre-stimulation of PSCs on the activation status of NK cells (Figure S12) [38].

## 4. Discussion

Clinical translation of hiPSC-derived cells requires management of their immunological consequences, locally and systemically, which may depend on the grafted cell type, site and mode of delivery [6,7,8]. The immunogenicity of hiPSCs and hiPSC-derived cells is thus a subject of research and controversy. Autologous hiPSC derivatives were expected to be tolerated by the host immune system. However, previous studies identified several factors impairing the acceptance of iPSC-derived cells in syngeneic animal hosts, caused by genetic alterations and aberrant gene expression associated with cellular immaturity or cultivation conditions [3,26,39,40]. Within the described project, the immunogenicity of hiPSC-derived renal cell types showing different developmental maturity levels was analyzed in autologous as well as in allogeneic in vitro setups.

The genes suspected to provide immune targets include ZG16, HORMAD1, SOX2, OCT4 and GATA3. Although ZG16 expression was detectable in PTCs and LT-PTCs and GATA3 expression was detectable in IMCs and PTCs, while hiPSCs expressed OCT4 and SOX2, none of these cell types triggered autologous T cell proliferation. Overall, autologous T cells did not respond against hiPSCs, IMCs, PTCs and LT-PTCs.

Surprisingly, allogeneic mismatched hiPSCs and hiPSC-derived renal cells were also not able to elicit T cell proliferation, even in the presence of preformed allogeneic-specific memory T cells. This was not the case for pUCs derived from the same donor as the hiPSC line used. In healthy individuals, the priming of naïve T cells against foreign HLA molecules occurs through heterologous immunity and prior exposure to allogeneic antigens, for example, due to blood infusion, pregnancy or organ transplantation [41]. Compared to unprimed naïve T cells, memory T cells only require HLA/peptide-TCR engagement without significant costimulatory signals and are less susceptible to conventional immunosuppressive drugs [42]. Thus, preformed allogen-specific memory T cells are the major obstacle in solid organ transplantation (SOT) and may likely play a role in allogeneic hiPSC-based CRT. This role was not supported by our allogeneic one-way MLR data. These data indicate, rather, that hiPSC-derived cells possess immune-privileged capacities and, in contrast to HLA-identical adult tissue cells, neither reactivate memory T cells nor induce naïve T cell proliferation. We hypothesized that active immune suppression by hiPSCs and hiPSC-derived cells may be responsible for this unexpected result. Indeed, when hiPSC-derived cells were exposed to allogeneic B cell-stimulated PBMCs, active immunomodulatory properties of hiPSCs, IMCs and PTCs were observed. These effects were not elicited by the more mature LT-PTCs or the terminally differentiated adult pUCs.

FOXP3^+^ regulatory T cells are essential for immune homeostasis and were shown under certain conditions to play a pivotal role in allogeneic mESC and hESC graft survival [43,44]. Furthermore, active polarization capabilities were described for mESC derivatives and hiPSC-derived RPECs, converting conventional T cells towards a regulatory phenotype [45,46]. Here we analyzed the potential of hiPSCs and hiPSC-derived renal cells to polarize naïve peripheral CD4^+^ T cells into iTregs. Co-culture experiments of allogeneic PBMCs with hiPSCs and hiPSC-derived renal cells, respectively, revealed no increased number of regulatory T cells within the CD4^+^ T cell population. Thus, T cell-mediated acceptance of hiPSCs and their renal progenitors was not based on iTreg conversion. However, in the presence of additional T cell stimulators, such as SEB or allogeneic B cells (data not shown), co-cultures with hiPSCs and IMCs showed increased numbers of T cells with a regulatory phenotype. The reason for the different outcomes could be explained by the varying concentrations of Interleukin 2 (IL-2). Besides TGFβ, the pro-inflammatory cytokine IL-2 is mandatory for the efficient polarization of iTregs due to the preservation of FOXP3 expression [47,48]. Additionally, IL-2 promotes the proliferation of CD4^+^CD25^+^ T cells [49]. Thus, a pro-inflammatory environment, mimicked in these experiments by polyclonal SEB stimulation, may be beneficial for the conversion and maintenance of peripheral naïve T cells into iTregs by hiPSCs and IMCs. Still, the underlying mechanism and the defined immunosuppressive reactivity of iTregs as well as that of natural Tregs (nTregs) needs to be elucidated.

It may be possible that different renal cell identities between pUCs and hiPSC-derived renal cells rather than maturation effects led to the different immune responses. pUCs are primary cells, which were cultivated to preferentially expand renal tubular epithelial cells and thus to be phenotypically close to the hiPSC-derived PTCs and LT-PTCs [22]. Morphology and transcriptome clustering clearly differentiated the various cell types and confirmed the phenotypic stability and functional maturation of LT-PTCs. This maturation of hiPSC-derived cells is a requirement to achieve functional equivalency for CRT; however, many hiPSC-derived cells are phenotypically immature.

The observed gradual maturation of renal cells was corroborated by immune phenotype changes. Transcriptome comparison of the hiPSC-lines with the parental pUCs confirmed decreased expression of MHC class I- and MHC class II-related genes, even under pro-inflammatory conditions. Renal differentiation induced a gradual up-regulation of MHC class I and the antigen-processing machinery. The key mediator of MHC class II, CIITA, which is constitutively expressed in antigen-presenting cells (APCs), can be transiently induced by inflammatory stimuli such as IFNγ in semi-professional APCs, such as primary PTCs during renal injury [50]. IFNγ is a potent inducer of MHC class II in semi-professional APCs and of MHC class I in somatic cells, affecting the immune phenotype of cells under pro-inflammatory conditions [51,52]. The IFNγ receptor genes IFNGR1 and IFNGR2 are expressed in hiPSCs and derived renal cells at similar levels (data not shown), but HLA-DR expression was only inducible in LT-PTCs. Overall, LT-PTCs showed a more mature immune phenotype compared to PTCs. Nevertheless, expression of MHC class I and II molecules in LT-PTCs was lower compared to somatic pUCs. In conclusion, allogeneic hiPSCs, IMCs, PTCs and LT-PTCs exhibit immunogenic potential but possess an unattractive immune phenotype and immune-suppressive capacities, which together lead to T cell tolerance.

Other than T cell responses, NK cell-mediated cytotoxicity may play a major role in CRT using hiPSC products [35,36]. Here, we revealed that autologous and allogeneic hiPSC-derived renal cells are recognized by NK cells. Autologous, as well as allogeneic pUCs, did not induce NK cell activation; thus, NK susceptibility is not related to allogenicity. Rather, it may be caused by reduced expression of MHC class I. Secretion of IFNγ by activated NK cells can stimulate IL-12 production by dendritic cells (DCs), which further promotes a T helper 1 polarization of naïve T cells due to T-bet induction [53]. Although IFNγ pre-treatment may protect against NK cell-mediated rejection due to upregulation of MHC class I expression, our study showed only a minor impact of IFNγ pre-stimulation of hiPSCs on the activation status of NK cells [37]. NK cells trigger an early immune event in organ transplantation, contributing to acute rejection, and should be controlled in clinical applications of autologous as well as allogeneic hiPSC-derived cells [54]. This control requires the analysis of human NK cell responses, preferentially in vitro co-cultivation systems, since common humanized mouse models show impaired reconstitution with NK and cytotoxic T cells, which is also seen in allogenized mouse models [55,56,57]. We opted to use fully humanized in vitro assays for sensitive detection of immune effects, which could perhaps also be used for individualized preclinical assessment of pre-transplant risk estimation. Nevertheless, although MLR is considered one of the oldest and most common in vitro functional tests used in transplant immunology, primarily, recipient T cell responses activated by the direct pathway through intact donor HLA alloantigens by APCs on the surface of the donor graft are measured [58,59]. In contrast, the indirect pathway occurs due to processed donor antigens presented by the recipient´s APCs and additionally plays a major role in acute and especially in chronic graft rejection. To fully cover indirect T cell alloreactivity within a MLR assay, either donor HLA peptides or donor lysate can be added to the co-culture samples [60].

Since the generation of patient-specific autologous hiPSC lines is cost- and time-intensive, the development of global stem cell haplobanks with accessible, quality-controlled and off-the-shelf HLA-typed hiPSC and hESC lines is therefore attractive to reduce risks associated with allogenic CRT [61]. Transplantation of PSC derivatives in non-human primates indicated that homozygous MHC-matched cardiomyocytes and RPECs showed improved engraftment compared to fully mismatched iPSC-derived cells [62,63]. However, it needs to be considered that minor histocompatibility, as well as blood group antigens, can lead to graft rejection and immune suppression will likely remain necessary [44]. Other strategies for tolerance induction are therefore being investigated and include the parallel overexpression of CTLA4 and CD274, co-transplantation of iPSC-derived regulatory dendritic cells or the generation of a universal hypoimmunogenic hiPSC line, lacking HLA-ABC and HLA-D expression but overexpressing inhibitory HLA-E [64,65,66]. Although these hypoimmunogenic hiPSCs could overcome allogeneic rejection barriers, the risk for tumorigenicity is increased due to immune evasion. Thus, this strategy requires additional safety measures by, for example, the inclusion of suicide genes.

## 5. Conclusions

In an era in which personalized medicine is taking front stage, stem cell-based therapies are becoming highly relevant. However, the immunogenicity of iPSCs and their progenies is a controversial topic. The therapeutic use of hiPSC-derived renal cells might be a game-changer to combat AKI- and CKD-associated kidney failure and overcome the high demand for kidney allografts. Our findings show the maturation-dependent immunogenicity of hiPSC-derived renal cells, which favors the use of immature tissue-specific cell types due to their strong immunomodulatory capacities even in an inflammatory tissue environment. However, advanced maturation of hiPSC-derived grafted cells may require control of arising immune competence and loss of immune tolerance. This switch must be considered in the preclinical assessment platforms of the therapeutic cell product. Furthermore, the immunomodulatory effects of immature hiPSC-derived precursor cells may favorably shape the local graft environment by reducing T cell activation. However, NK cell activation should be taken into consideration. Additionally, a putative risk of this low immunogenicity might be loss of control in case of viral infection or transformation. In vitro monitoring and assessment of the dynamic shift between immune privileges, immunomodulation and immune maturity stages could thus refine CRT. Finally, the applied example of renal differentiated cells may also be principally relevant for other solid mesoderm-derived tissues, but this will need comparative assessment.

## Data Availability

Additional data can be requested from the corresponding author.

## Supplementary Materials

The following are available online at www.mdpi.com/article/10.3390/cells11081328/s1, Figure S1: Expanding pUCs seeded on gelatin-coated cell culture plates 11 days after isolation, Figure S2: hiPSC-derived PTCs can be maintained, Figure S3: IMC- and PTC-specific markers are almost not present in hiPSCs, Figure S4: IMCs, PTCs, LT-PTCs and pUCs show expression of stage-specific proteins, while marker expressions of podocytes, distal tubules and collecting ducts are not detectable, Figure S5: Functional uptake analysis of 2-NBDG and FITC-albumin by hiPSCs, hiPSC-derived renal cells and pUCs, Figure S6: HLA-typing of healthy donors and patients with diabetic nephropathy revealed a maximum match in one out of six alleles with the hiPSC lines BCRTi004-A and BCRTi005-A, Figure S7: Pure populations of real naïve CD3^+^ T cells were sorted from full PBMCs using FACS, Figure S8: HLA-DR expression is elicited on CD4^+^ and CD8^+^ T cells after co-culture by allogeneic pUCs and not by hiPSCs and renal derivatives, Figure S9: Elevated TNF𝛼 release was detected after co-culture of pUCs with allogeneic PBMCs, Figure S10: Direct comparison of autologous and allogeneic T cell responses against hiPSCs, renal derivatives and pUCs revealed only immunogenic capacities of allogeneic pUCs, whereas hiPSC-derived stimulators did not elicit T cell proliferation, Figure S11: Generated and expanded B cells show an activated immune phenotype, Figure S12: IFNγ pre-stimulated hiPSCs elicit less NK cell activation, Table S1: List of primary antibodies, Table S2: Patient characteristics.
