# Supplementary material for: Human iPSC-Derived Renal Cells Change Their Immunogenic Properties during Maturation: Implications for Regenerative Therapies
Source: Cells. 2022 Apr 13;11(8):1328. doi: 10.3390/cells11081328 (PMC9032821; doi:10.3390/cells11081328)
Supplement: Supplementary file 1 [file cells-11-01328-s001.zip › cells-1660475-supplementary.pdf]

Supplementary Materials for"Human iPSC-derived renal cells change their immunogenic properties during maturation: Implications for regenerative therapies"

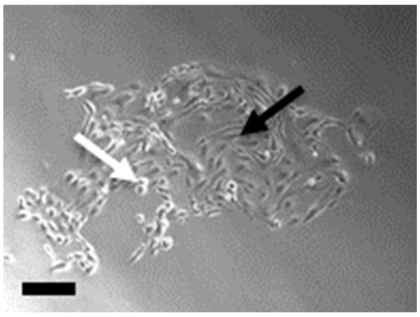

Figure S1: Expanding pUC seeded on Gelatin coated cell culture plates 11 days after isolation. As described by the original isolation protocol, dominantly two different morphological cell types with epithelial origin were detectable upon the expansion process [22]. White arrow indicates type 1 cuboid cells, black arrow refers to elongated spindle-shaped type 2 cells. Scale bar: 100 µm

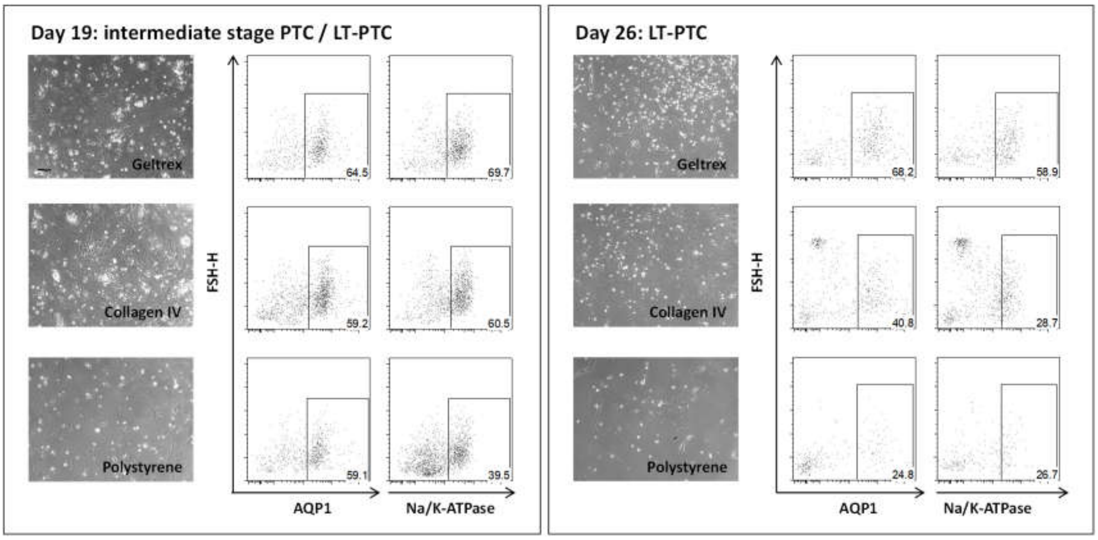

Figure S2. hiPSC-derived PTCs can be maintained *in vitro*. After terminal differentiation PTCs were cultivated for additional two weeks testing different coating reagents. Using flow cytometry analysis, Geltrex was revealed as the optimal matrix for the stable maintenance of PTCs to LT-PTCs based on AQP1 and Na/K-ATPase expression.

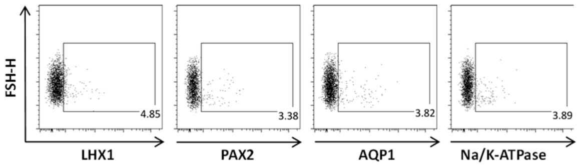

Figure S3. IMCs and PTCs specific marker are almost not present in hiPSCs.

Using flow cytometry the expression of IMCs and PTCs specific markers was tested in hiPSCs. Only background expression of the IMC markers PAX2 and LHX1 and PTC markers AQP1 and Na/K-ATPase was detectable in hiPSC.

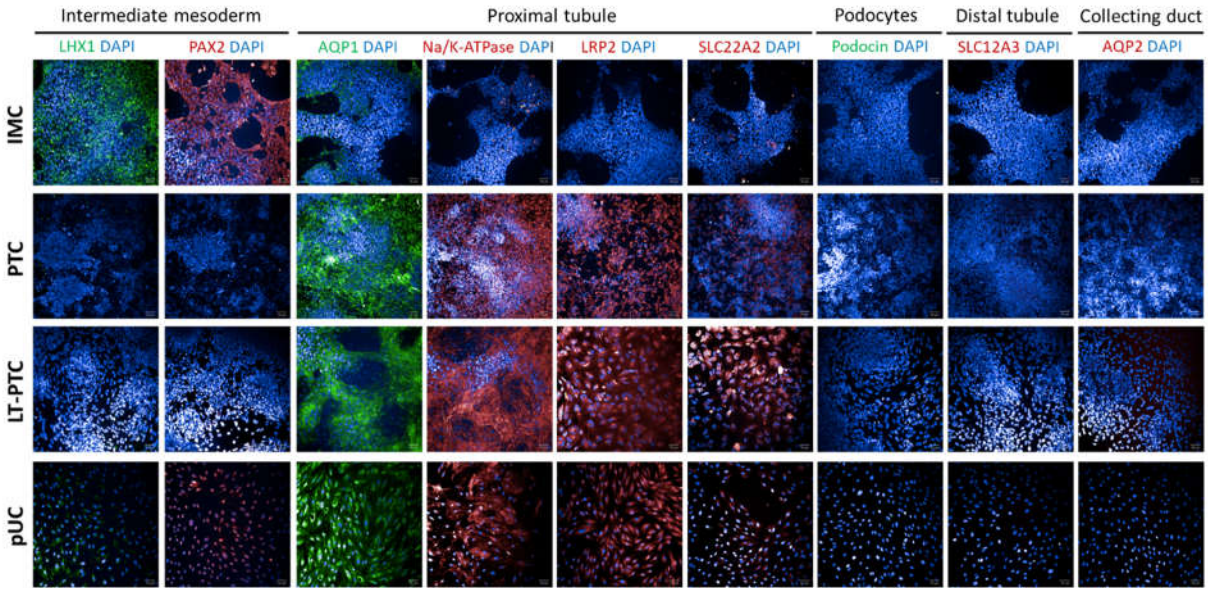

Figure S4. IMCs, PTCs, LT-PTCs and pUCs show expression of stage-specific proteins while marker expression of podocytes, distal tubule and collecting duct was not detectable. Immunofluorescence staining was performed to analyze IMCs, PTCs and LT-PTCs for stage-specific marker expression and the absence for markers of other parts of the nephron segments. Additionally, pUCs were included into the analysis to proof their proximal tubule origin. Background staining in the negative control appearing from the secondary antibody was subtracted. Scale bar is equivalent to 50 µm.

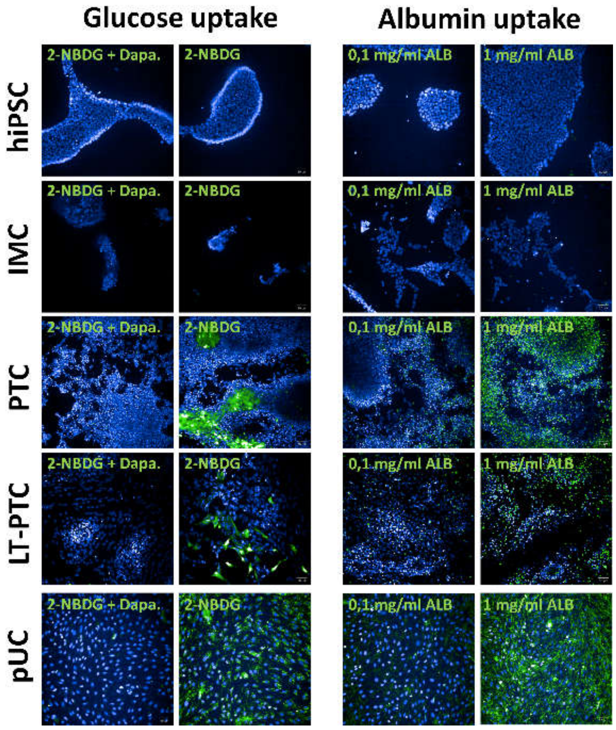

Figure S5. Functional uptake analysis of 2-NBDG and FITC-albumin by hiPSCs, hiPSC-derived renal cells and pUCs. Live cell imagining revealed the active uptake of the glucose analog 2-NBDG and FITC-albumin by PTCs, LT-PTCs and pUs and thus proved their proximal tubule identity. hiPSCs and IMCs did not show uptake capabilities of 2-NBDG or FITC-albumin Background staining in the negative control appearing from the secondary antibody was subtracted. Scale bar is equivalent to 50 µm.

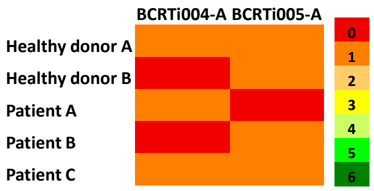

Figure S6. HLA-typing of healthy donors and patients with diabetic nephropathy revealed a maximum match in 1 out of 6 alleles with the hiPSC-lines BCRTi004-A and BCRTi005-A. From two healthy unrelated donors and 3 patients enrolled in the study HLA-types were available. Comparison with the HLA-A-, HLA-B- and HLA-DR-alleles, critical for kidney transplantation, of the hiPSC lines revealed a maximum overlap of one allele with the allogeneic donors.

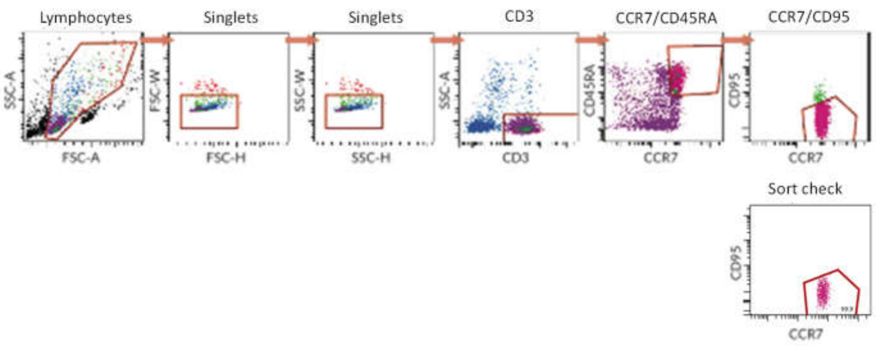

Figure S7. Pure populations of real naïve CD3⁺ T cells were sorted from full PBMCs using FACS. Isolated PBMCs from healthy donors were applied to a FACS device. The subpopulation of single CD3⁺CD45RA⁺CCR7⁺CD95⁻ cells was collected and subsequent sort check revealed a purity of up to 99.9%.

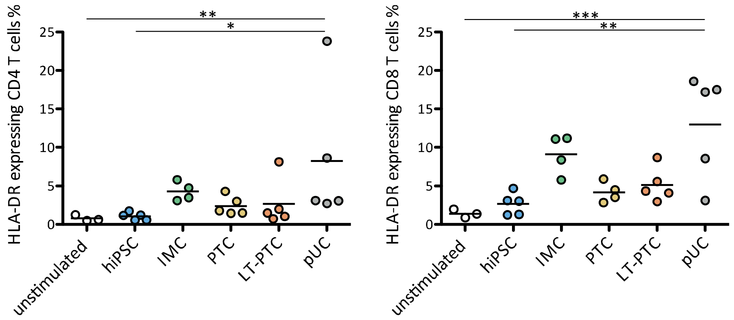

Figure S8. HLA-DR expression is elicited on CD4⁺ and CD8⁺ T cells after co-culture by allogeneic pUCs, and not by hiPSCs and renal derivatives. Expression of the late activation marker HLA-DR was assessed using flow cytometry on CD4⁺ and CD8⁺ T cells after a co-culture of 7 days with allogeneic hiPSCs, renal-derivatives and pUCs, respectively. Only allogeneic pUCs induced an increase of HLA-DR expression on CD4⁺ and CD8⁺ T cells, whereas hiPSCs and hiPSC-derived renal cells did not trigger activation of allogeneic T cells.

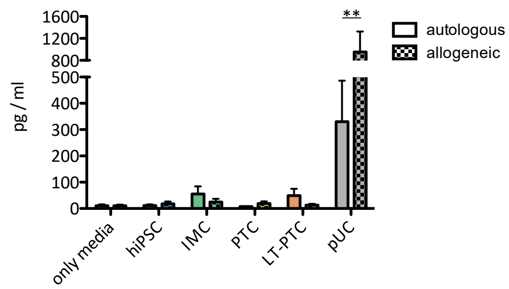

Figure S9. Elevated TNFα release was detected after co-culture of pUCs with allogeneic PBMCs. Supernatanta on day 3 of all experimental co-culture groups were analyzed for the accumulation of the pro-inflammatory cytokine TNFα via multiplex-assay. In comparison to autologous co-cultures, unstimulated controls and allogeneic co-cultures with hiPSCs and renal derivatives, only allogeneic pUCs induced increased TNFα release. Statistical analysis was performed using two-way ANOVA followed by Bonferoni´s post-test. **p < 0,01; ***p < 0,001.

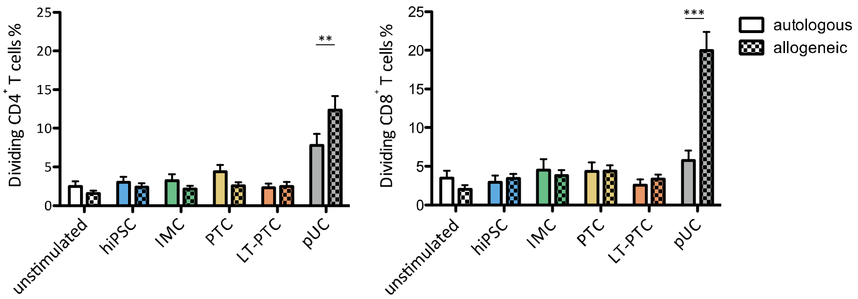

Figure S10. Direct comparison of autologous and allogeneic T cell responses against hiPSCs, renal derivatives and pUCs revealed only immunogenic capacities of allogeneic pUCs, whereas hiPSC-derived stimulators did not elicit T cell proliferation. Autologous and allogeneic $CD4^+$ and $CD8^+$ T cell proliferation was assessed after 7 days of co-culture with hiPSCs, renal derivatives and pUCs, respectively. After direct comparison between certain autologous and allogeneic T cell responses, only immunogenicity of allogeneic pUCs was observed. In contrast, although sharing the same HLA-type with the pUCs, hiPSCs and the different renal descendants did not provoke an allogeneic T cell response when comparing to autologous T cell responses as well as to unstimulated control samples. Statistical analysis was performed using two-way ANOVA followed by Bonferoni´s post-test. **p < 0,01; ***p < 0,001.

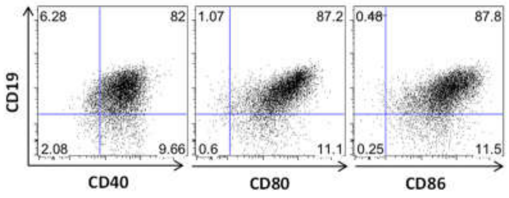

Figure S11. Generated and expanded B cells show an active immune-phenotype. Freshly thawn B cells were analyzed using flow cytometry. B cells were identified due to expression of CD19. $CD19^+$ cells were mainly co-expressing CD40, CD80 and CD86.

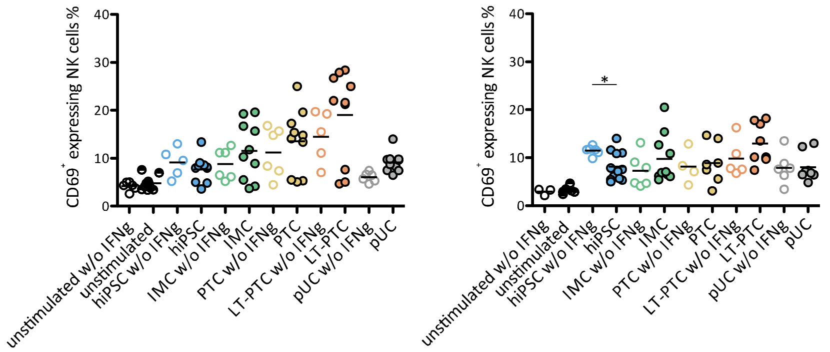

Figure S12: IFNγ pre-stimulated hiPSCs elicit less NK cell activation. Using flow cytometry the amount of CD69 expressing NK cells was determined after co-cultivation of PBMCs with unstimulated and IFNγ pre-stimulated autologous and allogeneic hiPSCs, IMCs, PTCs, LT-PTCs and pUC,s respectively. Comparison of NK cell activation status between unstimulated and IFNγ pre-stimulated samples revealed significant differences in the co-cultures of PBMCs with allogeneic hiPSCs. Statistical differences between certain unstimulated and IFNγ pre-stimulated samples were assessed using unpaired Mann-Whitney test. *p < 0,05.

Table S1: List of primary antiodies

Antigen	Company	Catalogue #
AQP1	Novus Biologicals	NB600-749
AQP1	Proteintech	20333-1-AP
AQP2	Novus Biologicals	NB110-74682
CCR7	BioLegend	353206
CD3	BioLegend	300420
CD3	BioLegend	317324
CD4	BioLegend	344608
CD8	eBioscience	14-0086-80
CD25	BD Biosciences	555432
CD40	BioLegend	334306
CD45RA	BioLegend	304146
CD56	Beckman Coulter	A07788
CD69	BioLegend	310930
CD80	BD Biosciences	557227
CD86	BD Biosciences	555666
CD95	BioLegend	305622
CTLA4	BD Biosciences	560938
FOXP3	BD Biosciences	560082
HLA-ABC	BioLegend	311418
HLA-DR	BioLegend	307610
LHX1	Novus Biologicals	NBP2-01926
Live/Dead Blue	Invitrogen	L23105
LRP2	Abcam	ab76969
Na/K-ATPase	Abcam	ab58475
NPHS2	Abcam	ab50339
PAX2	Life Technologies	71-6000
SLC12A3	Novus Biologicals	NBP1-59699
SLC22A2	Abcam	ab92458

Table S2: Patient characteristics

Patient characteristics	Occurrence
Number of patients	6
Average Age ± SD	43 ± 14,18
Gender (f / m)	3 / 3
Diagnosis Diabetes mellitus Type 1 (Juvenile / Adult)	3 / 1; 2 unknown
Need of dialysis	1 / 5 (hemodialysis); 1 unknown
